# Prevalence of oral mucocele among outpatients at saveetha dental hospital, india.

**DOI:** 10.6026/973206300161013

**Published:** 2020-12-31

**Authors:** Shalini Sathiyamoorthy, S Gheena, Ravindra Kumar Jain

**Affiliations:** 1 Saveetha Dental College, Saveetha Institute of Medical and Technical Sciences, Saveetha University, Chennai 77, India

**Keywords:** Mucocele, benign lesion, upper lip, minor salivary gland

## Abstract

Oral mucoceles are the most common benign minor salivary gland lesions. It is of interest to document the prevalence of oral mucocele among outpatients at the Saveetha Dental Hospital, India. We used patient data (12 case records) with mucocele occurrence for this analysis. Data included age, gender, diagnosis, lesion duration and relevant dental history. Data shows that oral mucocele were seen predominantly in males (66%) when compared to females (34%). The most affected site in the oral cavity was the lower lip (58%). Thus, data shows that oral mucocele was predominantly seen in males compared to females. Data also shows that the lower lip is often affected.

## Background

Mucocele is a soft tissue benign lesion of the minor salivary gland characterised by a cavity filled with mucous due to mucous extravasation. These are the most common cystic lesions affecting the oral mucosa [1]. Whole saliva is mainly composed of fluid produced by major and minor salivary glands [2]. This can appear in the oral cavity, gallbladder, appendix, paranasal sinuses and lacrimal sac [3,4]. The term mucocele is derived from the latin word mucus and cocele means cavity [5]. Mucocele is the 17th most common salivary gland lesion seen in the oral cavity [6]. This is the result of accumulation of liquid or mucoid material due to the alteration in the minor salivary gland which causes limited swelling [7,8]. It is clinically characterised by single or multiple, painless, soft, smooth, spherical, translucent, fluctuant, nodule, which is usually asymptomatic [9]. Oral mucoceles are usually dome shaped enlargements with intact epithelium [10]. They are classified as extravasation or retention type [11]. Toxins in cigarette smoke contain numerous free radicals causing tissue damage in the oral cavity [12]. Salivary dysfunction, reduced salivary PH and salivary hyperglycaemia provide a potential substrate for fungal growth [13]. Tumours that arise after six month are called metachronous tumours [14,15]. Progressive deposition of calcified masses originating from the root may increase the susceptibility of pulp stone formation [16]. Multinucleated giant cells epithelioid cells and macrophages and its derivatives are the most characteristic histological features that dictate the granuloma feature [17,18]. The extravasation type is a pseudocyst without defined walls and is caused due to mechanical trauma to the excretory duct of the gland leading to the rupture with consequent extravasation of mucin into the connective tissue stroma and is seen frequently on lower labial mucosa, buccal mucosa and retromolar area [19]. The retention type is less common than extravasation, usually upper lip, hard palate, floor of mouth and maxillary sinus [20,21]. The lesion can be located directly under the mucosa (superficial mucocele), in the upper submucosa (classic mucocele), or deep corium (deep mucocele) [22]. Interactions between epithelial cells and the connective tissue play a major role in many biological processes [23, [24]. The oral mucocele located on the floor of the mouth are termed as ranula, which usually arises in the body of the sublingual gland and occasionally in the duct of rivinus or in the Wharton's duct [6]. Ranulas are considered as a variant of mucocele and the name is derived from the typical swelling that resembles the air sacs of the frog "Rana tigrina" [25,26]. A ranula manifests as a cup shaped fluctuant bluish swelling on the floor of mouth and tends to be larger than mucocele located in the other regions of the mouth [27]. Early detection and prompt diagnosis can lead to better prognosis and help in the implementation of successful clinical treatment [26,28]. Therefore, it is of interest to document the prevalence of oral mucocele among the outpatient population at Saveetha Dental College.

## Methodology

The retrospective study was conducted in a hospital setting. The available data with similar ethnicity was collected from a particular geographical location. The trends in other locations were not assessed in this study setting. The Institutional ethical committee gave ethical approval. The ethical approval number is SDC/SIHEC/2020/DIASDATA/0619-0320.The data was collected within a time period of June 2019 to April 2020.Case sheets were reviewed and cross verification done by another examiner to avoid errors. To minimise the sampling bias all-available data was included and no sorting process was done. All samples that were diagnosed with mucocele at that particular time were considered as internal validity and a prescriptive pattern was followed to analyse the external validity. Patient Data on Mucocele occurrences in an outpatient population who visited a Dental college was collected. All the records like patients ID, age, gender, diagnosis, lesion duration, and relevant dental history were collected. This data was entered in excel sheet and tabulated and SPSS importing was done including analysis by chi Square test. Patients who were not diagnosed with mucocele were considered as variables. Statistical test used was chi - Square test and the software used is SPSS.

## Results and Discussion:

The total sample size of our study was 12 patients in which 66% were male and 34% were female. There was a significant preference for the lower lip site in the oral cavity for 7 patients followed by Upper lip for 3 and tip of the tongue for 2. The causative factor for the oral mucocele occurred due to trauma in 5 subjects and with no history in 7 subjects. In this study we observed that oral mucocele was predominantly higher in males (66.4%) than females (34%). The most affected site in the oral cavity was lower lip (58%).

Many epidemiological studies regarding the occurrence of oral mucoceles have given a vast amount of information. Different authors showing a wide range of variety have obtained different results. Study by Chandramani et al. [29] conducted hospital based retrospective study. Institutional ethics committee gave ethical approval. In sample size of 58 cases of oral mucocele male were predominantly affected more (51.72%) than female (48.2%). Most commonly affected sites were lower lip (36.2%) in males. Causative factors for oral mucocele with no cause were (72.41%), lip biting (22.41%) and trauma (5.18%). In this study they concluded that the lower lip was the most commonly affected site. Known data is in consensus with the present study (Figure 1 -see PDF). Tegginamani et al. [30] conducted a Hospital based Retrospective study. In a sample size of 50 cases of oral mucoceles, male were predominantly affected more (78%) than females (22%). Oral mucoceles were highly prevalent in the age group of 15 - 19 years (34.48%). Most commonly lower lip was involved (90%) followed by floor of mouth (6%) and ventral surface of the tongue (4%). The etiology of history of trauma was present in 76% of the cases mostly lip biting. Previous literature was in consensus with the present study (Figure 2 - see PDF). Jain et at. [31] conducted a hospital based retrospective study. In the sample size of 36 cases of oral mucoceles in which males were predominantly affected more (63.8%) when compared to females (36.1%). Most commonly the lower labial mucosa lateral to the midline was involved (94.44%). History of trauma was seen in (77.7%) followed by no causes (22.2%). Previous literature was in consensus with the present study.

## Conclusion

We show that oral mucocele was predominantly seen in males compared to females. Data also shows that the lower lip is often affected. More data is needed to validate the current observation.
